# U.S. Navy’s Response to a Shipboard Coronavirus Outbreak: Considerations for a Medical Management Plan at Sea

**DOI:** 10.1093/milmed/usaa455

**Published:** 2020-11-30

**Authors:** Diego Vicente, Ryan Maves, Eric Elster, Alfred Shwayhat

**Affiliations:** Department of Surgery, Naval Medical Center San Diego, San Diego, CA 34800 San Diego, CA 92134, USA; Department of Surgery, Uniformed Services University of the Health Sciences & the Walter Reed National Military Medical Center, Bethesda, MD 4301 Bethesda, MD 20814, USA; Department of Medicine, Naval Medical Center San Diego, San Diego, CA 34800 San Diego, CA 92134, USA; Department of Medicine, Uniformed Services University of the Health Sciences, Bethesda, MD 4301 Bethesda, MD 20814, USA; Department of Surgery, Uniformed Services University of the Health Sciences & the Walter Reed National Military Medical Center, Bethesda, MD 4301 Bethesda, MD 20814, USA; Department of Medicine, Naval Medical Center San Diego, San Diego, CA 34800 San Diego, CA 92134, USA; Department of Medicine, Uniformed Services University of the Health Sciences, Bethesda, MD 4301 Bethesda, MD 20814, USA

The authors observe with interest the ongoing management of the 2019 coronavirus (COVID-19) pandemic and its impact on the U.S. Navy. The Navy is in a unique position of needing to balance operational readiness with the management of a highly contagious and morbid respiratory virus while working in close proximity and in confined spaces at sea or in preparations for sea. The widely accepted components of pandemic control include social distancing, isolation of sick patients, and quarantine of exposed persons. These components are almost entirely inconsistent with life at sea on a warship. The tight quarters and close interactions of over 5,000 personnel onboard a U.S. Navy aircraft carrier (CVN) make prevention of respiratory virus transmission unusually difficult. Any attempts to evaluate, manage, isolate, and transport infected personnel within or off the ship would be made more challenging by operational requirements as well as limited ambulatory space, lavatory areas, berthing spaces, mess halls, supplies, medevac capabilities, and medical capacity. Further, unlike the U.S. Navy’s prior experience in managing shipboard noncritically ill patients with gastrointestinal or respiratory infections,^1^ the management of an infectious respiratory outbreak with numerous seriously ill patients aboard a large U.S. Navy vessel has not been defined to the authors’ knowledge before COVID-19. Although the challenges seem overwhelming, the U.S. military has extensive experience in infectious disease, preventive medicine, and providing critical care in austere environments.^2^ Herein, the authors apply the known challenges of COVID-19 management to describe a potential model of a shipboard outbreak and illustrate the clinical strain on existing ship’s resources as well as describe the means necessary to mitigate the morbidity and mortality of such an outbreak.

A respiratory virus outbreak aboard a CVN raises particular concern for an infectious disease with a relatively high transmission rate, high resource utilization, and mortality risk. The 1918 pandemic H1N1 influenza virus is a well-documented example of a pandemic respiratory virus with a high mortality rate (1%) for 20- to 40-year-old patients.^3^ The transmission rate of an H1N1 influenza virus, however, was relatively low with a basic reproduction number (*R*_0_) of 1.0 to 1.5, and transmission occurred primarily in symptomatic patients.^4^ This means that, under standard circumstances in the general population, every symptomatic H1N1 patient will infect one other person. While COVID-19 is associated with a lower mortality rate (around 0.2% in the 20- to 40-year-old age group),^5^ its *R*_0_ is 2.2 to 2.7 and virus transmission can occur via asymptomatic (or presymptomatic) carriers.^6,7^ This means that every patient with COVID-19 may infect at least two other people, resulting in exponential transmission as compared to the more linear transmission of an H1N1 virus.

Shipboard life is not a standard environment, and transmission of respiratory viruses occurs more frequently.^8^ Prior respiratory virus outbreaks on naval vessels report attack rates between 12% and 86%, meaning the majority of a ship’s crew may be easily infected.^8^ The attack rate for pandemic influenza A (H1N1) 2009 virus aboard a U.S. Navy vessel with approximately 2,000 personnel was reported to be 37%.^1^ Specifically for COVID-19, the attack rate reported aboard the Diamond Princess was 19% (*n* = 712) with 46% (*n* = 331) of these patients being asymptomatic or presymptomatic at the time of testing.^9^

Recently, publicly available data regarding the well-documented outbreak of COVID-19 onboard the USS Theodore Roosevelt (CVN-71) on deployment in April 2020 have become available. Approximately 1,000 sailors onboard were known to have been infected during this; of these infected sailors, several required hospitalization at the U.S. Naval Hospital Guam for severe disease, with one sailor dying of the disease. Antibody testing of a representative 382 crew members (known in epidemiology as a “convenience sample”) indicated that nearly 20% of infected sailors lacked symptoms but would have been capable of transmitting the virus, a recipe for unwitting spread onboard a cramped vessel.^10^ Although the great majority of CVN-71 sailors with infection experienced mild disease, we cannot overlook the non-inconsequential number who required hospitalization (usually for low oxygen levels associated with pneumonia) as well as the sailor who died. One death out of a thousand is lower than the estimated 0.5% to 1% mortality rate of COVID-19 in the general population, but we must consider that the number of deaths aboard CVN-71 could have been higher without the expert critical care support of the U.S. Naval Hospital Guam. Indeed, the reported morbidity and medical requirements at the time of the CVN-71 outbreak would have quickly exhausted the ship’s medical capabilities.^6,11–13^ Ultimately, and similar to military medicine’s objectives in trauma care, our goal should be to have zero preventable deaths because of infectious disease.^14^

How, then, could a deployed warship manage an outbreak with limited patient transport capabilities and limited portside support? The authors constructed the following model, considering the available information at the time of the CVN-71 COVID-19 outbreak, to guide potential strategies in future outbreaks. An attack rate of 40% for a respiratory virus will be assumed to account for ship conditions and prolonged exposure over several months at sea without the possibility of evacuation. Given that rigid health screenings are in place to favor a healthy population aboard a CVN, the low end of CDC-reported medical needs for the 20- to 40-year-old population will be assumed. This includes an 83% rate of symptomatic patients of which 14% could require hospitalization and 2% would require intensive care unit (ICU) management.^13^ Based on the available data, up to 50% of ICU patients would require ventilator support.^15^ Additional considerations would be required for the highest risk patients aboard a CVN, namely patients over the age of 50 years and those whose comorbidities would increase morbidity, mortality, and required medical resources.^13^ The authors recognize that this age-restricted model is hypothetical; however, with adjustments to the attack rate and morbidity rate, the proposed management strategy could be broadly applicable.

Given the hypothetical parameters outlined above, a COVID-19 outbreak aboard a Nimitz-class CVN with approximately 5,000 personnel would result in 2,000 infected patients over the course of several months at sea. Of these, 83% (*n* = 1660) would develop symptoms and another 14% (*n* = 232) would require hospitalization for supportive care, potentially including supplemental oxygen. Subsequently, 2% (*n* = 33) of infected patients with symptoms would require ICU-level care, and up to 50% (*n* = 16) of these ICU pati-ents could require endotracheal intubation and mechanical ventilation.

As seen in medical systems around the world, this sudden surge in sick patients would rapidly overwhelm the CVN’s medical capabilities. The Nimitz-class carrier medical department is composed of 40 to 60 personnel with varying levels of ICU experience as shown in Table I. Care is provided to patients with capacity for 3 ICU beds, 15 hospital beds, 1 operating room, 1 emergency room, and multiple smaller clinical spaces. The ICU-level care is run by the ship’s surgeon in coordination with the ship’s nurse and nurse anesthetist, and the authors have each witnessed the outstanding care that can be provided at sea by this team. Even with onboard support from other providers, this team would be significantly limited in the management of multiple critically ill patients, given that there are typically only four ventilators aboard a Nimitz-class CVN. Further, the resources to maintain these ventilated patients would be quickly exhausted without additional equipment and medical personnel support.

**TABLE I.** Medical Personnel aboard a U.S. Navy Nimitz-class CVN and Potential Roles in the Management of a Respiratory Virus Outbreak While at Sea

	CVN medical capabilities for a respiratory virus outbreak			
	ICU	Triage	Nonvirus patients	Augmentation required
1 Senior medical officer	X^a^	X	X	
1 General surgeon	X			X^b^
1 Nurse anesthetist	X^a^			
1 Family medicine physician	X^a^	X	X	
1 Psychologist		X	X	
1 Medical administration officer		X	X	
1 Radiation health officer		X	X	
1 Physical therapist		X	X	
1 Physician assistant	X^a^	X	X	
1 Nurse	X^a^			X
3-5 Flight surgeons	X^a^	X	X	
1-2 Independent duty corpsmen		X	X	
40-50 Hospital corpsmen^c^	X^a^	X	X	X^d^
Dental department		X	X	

Abbreviations: CVN, aircraft carrier; ICU, intensive care unit.

a ICU experience varies widely.

b Augmentation with ICU physician.

c Corpsmen including lab techs, radiology techs, preventive medicine techs, surgery techs, and biomedical techs.

d Additional preventive medicine techs, lab techs, and respiratory therapists would be required.

Hence, the following measures should be implemented early in an outbreak to mitigate the situation and keep the ship fighting: (1) early recognition mechanisms by preventive medicine technicians to detect an uptick in symptoms or illnesses signaling the beginning of an epidemic, (2) proactive involvement of shore-based preventive medicine teams to assist in developing contact tracing and recommend quarantine measures, (3) ship-wide education and appropriate distribution of available personal protective equipment and cleaning supplies, (4) ship zoning to isolate and manage patients with suspected infection and quarantine enforcement for mildly symptomatic patients, (5) risk stratification of ship’s crew to identify and quarantine or evacuate the highest risk nonessential personnel, (6) up-scaling existing transport systems for resources and patient movement, (7) consideration for temporary mobilization of nonessential carrier air wing components off the ship to increase hangar deck space to accommodate additional clinic and berthing space in a well-ventilated area, (8) establishment of telehealth between medical teams and isolation berthing spaces, (9) initiation of buddy aid support systems in the isolation berthing spaces, (10) designation and staffing of at least two clinical spaces to separately address patients with respiratory viral symptoms away from patients with other clinical concerns, (11) rapid inventory of available supplies and engagement of supply chain, and (12) import of shipboard testing for the respiratory virus in question.

In a situation where community spread is ongoing aboard the CVN despite the above measures, the higher authority may consider the following: (1) continue with mission of the ship with a plan to medevac patients as conditions allow, (2) augment the ship’s medical staff with additional teams and supplies to help manage the impending surge in patient volume, or (3) to go “offline” and pull into port. Per the model described above, additional resources to care for patients at sea would include the following: (1) creation of 3 separate critical care teams including 2 additional ICU doctors to augment the Ship’s Surgeon, 2 additional ICU nurses to augment the Ship’s Nurse, as well as at least 3 respiratory therapists to assist with ventilators and noninvasive oxygen support, (2) assignment of additional medical providers and corpsmen to support ICU teams as appropriate, (3) at least 16 additional portable transport ventilators and supporting equipment such as tubing and filters, (4) medications to support critically ill patients (e.g., sedatives, paralytics, anticoagulants), (5) supplemental oxygen delivery equipment such as nasal cannula, (6) supplies to establish arterial and intravenous lines, (7) monitoring equipment to follow cardiovascular and pulmonary markers, (8) extended lab capabilities to both manage ICU patients (e.g., arterial blood gases, coagulation studies) and also to test for infection, (9) transport systems to achieve delivery of these supplies and to evacuate personnel who have exceeded the medical capacity of the ship, and (10) augmentation of Nurse Anesthetist, Physician Assistant, and Psychologist to provide redundancy for sedation procedures, triage, and mental health, respectively. This is, in summary, setting up a temporary 16-bed ICU aboard a CVN. Additional supplies and personnel may be required over time as infection rates and contamination affect medical crew and equipment. Further, a consideration for maintaining capabilities for the management of a mass casualty event while at sea need to be taken into account. Formal evaluation of ongoing U.S. Navy shipboard outbreaks as well as subsequent simulations and models may aid in the development of appropriate response teams.

Since the start of the conflicts in Iraq and Afghanistan, the U.S. military has reported improved survival rates of combat-wounded despite increasing severity of injury from complex blast injury and the management of these casualties in austere environments.^2^ Among the advancements in care that produced these outcomes, the introduction of Critical Care Air Transport Teams has demonstrated the capacity to safely manage patients requiring blood product resuscitation, as well as cardiovascular and ventilator support across thousands of miles of air travel. These teams serve as a model for the rapid mobilization of the military’s existing resources to care for the sickest patients. A maritime Critical Care Air Transport Teams, using naval-capable aircraft such as the V-22, could be considered to help manage a shipboard viral respiratory outbreaks at sea, with rapid delivery of established Authorized Medical Allowance List packs, preventive medicine teams from the regional Navy Environmental and Preventive Medicine Unit, and trained personnel to augment the ship’s clinical capabilities, as well as permitting for medevac of critically ill patients from deployed ships once stabilized. In addition, medical care could be enhanced with telemedicine between shipboard personnel and shore-based specialists. Finally, existing ship vaccination programs could be adapted as the vaccines to manage these outbreaks become available.

Coronavirus of 2019 is not the first pandemic in our Navy’s history, and we would be remiss to assume that it will be the last. The general strategies for shipboard containment that we describe here may be broadly applicable to a variety of contagious diseases, from a novel strain of pandemic influenza to viral hemorrhagic fevers. Similarly, a capacity for augmenting critical care capability at sea, both shipboard and en route care, with dedicated and mobile teams can expand the reach of Navy Medicine to care for the fleet in the deployed environment. The viewpoints in this article come from two prior Ship’s Surgeons, a prior Senior Medical Officer, an infectious disease and critical care specialist, and a prior carrier air wing flight surgeon, all of whom have combat deployment and shipboard experience.

The authors recognize that the described model will not be perfectly applicable to all respiratory virus outbreaks as it was estimated using preliminary data (initially drafted in April 2020) and a literature review specific to COVID-19. Further, the authors recognize that medical resources vary significantly between ships and that the majority of U.S. Navy vessels’ medical capabilities are limited to an independent duty corpsmen. Extension of the proposed model to manage a COVID-19 outbreak aboard a ship with fewer personnel and resources than a CVN in an austere environment would largely be focused on containment, supportive care, and acquisition of additional resources. Beyond the proposed model, ongoing investment in prevention of COVID-19 outbreaks is key. As this pandemic has evolved, prevention methods have dramatically decreased COVID-19 spread in military and civilian populations. Specifically for ships, ongoing social distancing, “bubble-to-bubble” interactions, restriction of movement and COVID-19 testing for new personnel, mask use, handwashing, frequent surface cleaning (“clamp downs”), and daily relevant COVID-19 updates (NRTP_4-2.10 SHIPBOARD QUARANTINE AND ISOLATION) are key to prevention of COVID-19 outbreaks at sea.

With these models’ limitations in mind, formal epidemiologic modeling by the Navy Environmental and Preventive Medicine Unit and Navy and Marine Corps Public Health Center of ship-based contagion and development of shore-based response teams for shipboard viral outbreaks could mitigate causalities in future events and maintain operational readiness at sea. In so doing, Navy Medicine can maintain its mission of increasing lethality of our forces by eliminating preventable deaths with “Medical Power for Naval Superiority.”
